# Vesicle trafficking and vesicle fusion: mechanisms, biological functions, and their implications for potential disease therapy

**DOI:** 10.1186/s43556-022-00090-3

**Published:** 2022-09-21

**Authors:** Lele Cui, Hao Li, Yufeng Xi, Qianli Hu, Huimin Liu, Jiaqi Fan, Yijuan Xiang, Xing Zhang, Weiwei Shui, Ying Lai

**Affiliations:** National Clinical Research Center for Geriatrics, State Key Laboratory of Biotherapy and Collaborative Innovation Center of Biotherapy, West China Hospital, Sichuan University, Chengdu, China

**Keywords:** Vesicle formation, Vesicle transport, Vesicle fusion, Fusogenic proteins, Disease therapy

## Abstract

Intracellular vesicle trafficking is the fundamental process to maintain the homeostasis of membrane-enclosed organelles in eukaryotic cells. These organelles transport cargo from the donor membrane to the target membrane through the cargo containing vesicles. Vesicle trafficking pathway includes vesicle formation from the donor membrane, vesicle transport, and vesicle fusion with the target membrane. Coat protein mediated vesicle formation is a delicate membrane budding process for cargo molecules selection and package into vesicle carriers. Vesicle transport is a dynamic and specific process for the cargo containing vesicles translocation from the donor membrane to the target membrane. This process requires a group of conserved proteins such as Rab GTPases, motor adaptors, and motor proteins to ensure vesicle transport along cytoskeletal track. Soluble N-ethyl-maleimide-sensitive factor (NSF) attachment protein receptors (SNARE)-mediated vesicle fusion is the final process for vesicle unloading the cargo molecules at the target membrane. To ensure vesicle fusion occurring at a defined position and time pattern in eukaryotic cell, multiple fusogenic proteins, such as synaptotagmin (Syt), complexin (Cpx), Munc13, Munc18 and other tethering factors, cooperate together to precisely regulate the process of vesicle fusion. Dysfunctions of the fusogenic proteins in SNARE-mediated vesicle fusion are closely related to many diseases. Recent studies have suggested that stimulated membrane fusion can be manipulated pharmacologically via disruption the interface between the SNARE complex and Ca^2+^ sensor protein. Here, we summarize recent insights into the molecular mechanisms of vesicle trafficking, and implications for the development of new therapeutics based on the manipulation of vesicle fusion.

## Introduction

In eukaryotic cells, vesicle trafficking dependent signal transduction occurs via exchange of the content between membrane-enclosed organelles. Endoplasmic reticulum (ER), Golgi apparatus, endosomes, multivesicular body, lysosome, and plasma membrane are well characterized vesicle trafficking organelles [1]. Cargo containing-vesicle trafficking is highly specific and dynamic, which is regulated by multiple proteins that are conserved throughout eukaryotic evolution [2, 3]. Vesicle trafficking initiates from vesicle formation via membrane budding, followed by vesicle transport among intracellular organelles, and is accomplished by vesicle fusion with the target membrane [4]. Up to one third of all proteins in eukaryotic cells involve in these vesicle trafficking pathways. These events are orchestrated by mutiple proteins and protein complexes, including coat proteins (such as coat protein complex II (COPII), COPI, and clathrin), small GTP-binding proteins, tethering proteins, and fusogenic proteins [3, 5–11].

Dysfunctional vesicle trafficking could lead to the development of a wide range of diseases, for example in the nervous system, dysregulation of synaptic vesicle fusion in presynapse leads to neurodegenerative disease; in the respiratory system, abnormal secretory activity of airway epithelial cells results in several respiratory disorders, such as asthma and cystic fibrosis. Here, we present a general working model for how vesicles are transported between membrane organelles, and propose a potential new therapeutic strategy for diseases that are related to vesicle fusion.

## Vesicle formation

**Fig. 1:**
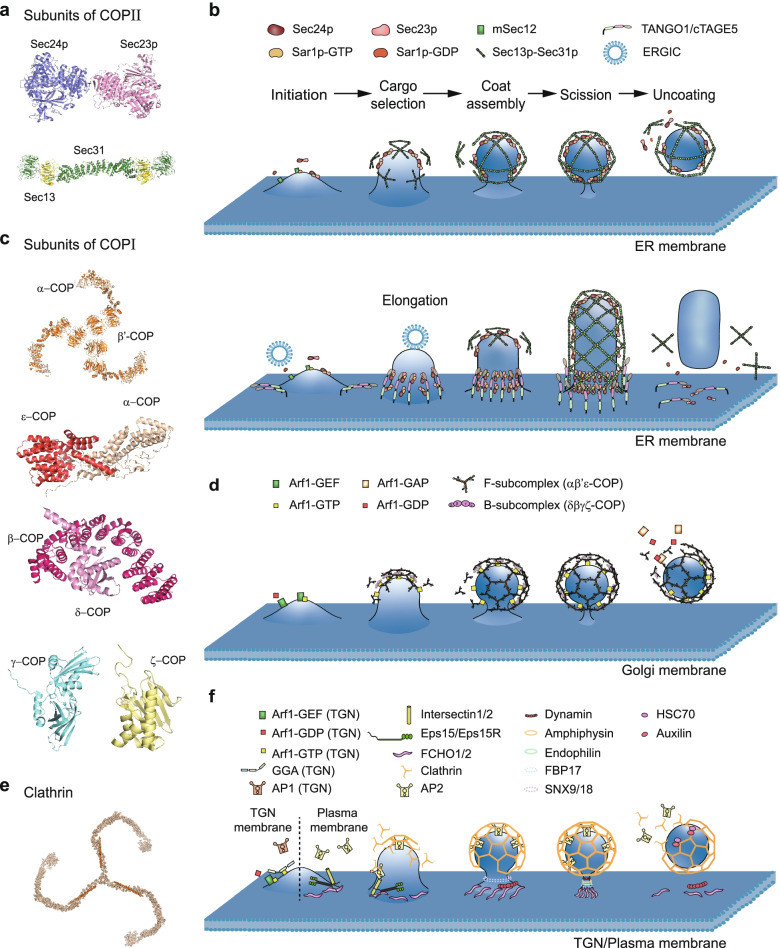
The coat protein mediated vesicle formation. **a** Crystal structure of the Sec23p-Sec24p subcomplex in *Homo sapiens* (PDB ID: 3EG9) [12] and cryo-EM structure of the Sec13-Sec31 subcomplex in *Saccharomyces cerevisiae* (PDB ID: 4BZK) [13]. **b** COPII-coated vesicles formation. Upper panel, COPII-coated vesicles derived from the ER exit site (ERES). Lower panel, formation of a mega-COPII-coated carrier at the ER. **c** Crystal structure of αβ’-COP subcomplex in *Saccharomyces cerevisiae* (PDB ID: 3MKQ) [14], αε-COP subcomplex in *Bos taurus* (PDB ID: 3MKR) [14], βδ-COP subcomplex in *Chaetomium thermophilum* (PDB ID: 5MU7) [15], the γ-COP subunit in *Homo sapiens* (PDB ID: 1R4X) [16] and NMR structure of the ζ-COP subunit in *Homo sapiens* (PDB ID: 2HF6) [17] of the COPI complex. **d** COPI-coated vesicles formation. COPI-coated vesicles appear in retrograde transport from the Golgi apparatus to the ER and between the Golgi cisternae. **e** Cryo-EM structure of clathrin triskelia in *Bos taurus* (PDB ID: 3IYV) [18]. **f** CCVs formation from TGN (on the left of the dotted line) and plasma membrane (on the right of the dotted line). The copyright permission of panels b, d, and f are from [7]

In mammalian cells, vesicle formation is a complex and delicate process achieved by trapping cargo molecules into carriers on the intracellular membrane compartments. Several intracellular organelles are involved in this process, such as ER, Golgi, and plasma membrane. Different coat proteins mediate different budding events, by shaping of transport vesicles and selecting desired cargo molecules through direct or indirect interactions. COPII-, COPI- and clathrin-coated vesicles (CCVs) make up most of intracellular trafficking vesicles. They share conserved molecular rationale for vesicle formation, although the involved molecules are different from each other. In this review, we summarize the studies for the three well defined pathways of vesicle formation that are mediated by COPII, COPI and clathrin (Fig. 1, Table 1).

**Table 1:** Classic proteins involved in COPII-, COPI-, and clathrin-mediated vesicle formation

	**COPII**	**COPI**	**Clathrin**	
Location	ER	*cis-*Golgi	TGN	Plasma membrane
Initiation protein	mSec12 Sar1p-GTP	Arf1-GTP Arf1-GEF (GBF1)	Arf1-GTP Arf1-GEF	FCHO1/2 intersectin1/2 eps15/eps15R
Adaptor protein	Sec24p Sec23p p24 TANGO1/cTAGE5	Arf1 Arf1-GEF	AP1 GGAs	AP2
Cargo molecule	Collagens chylomicron Proteins containing diphenylalanine motif	Proteins containing di-lysine motifs (K(X)KXX), or RXR motifs	Proteins containing YXXΦ, LL motifs, or (D/E)XXXL(L/I) motif	Proteins containing YXXΦ, LL motifs, or (D/E)XXXL(L/I) motif
Coat protein	Sar1p Sec23p Sec24p Sec13p Sec31p	α-COP β′-COP β-COP δ-COP γ-COP ζ-COP	Clathrin	Clathrin
Membrane scission protein	Sec23p Sar1p-GTP	Arf-GAPs PLD2 BARS	Dynamin Amphiphysin	Dynamin Endophilin Amphiphysin FBP17 SNX9/18
Uncoating protein	Sec23p Sar1p-GTP	Arf1-GTP Arf1-GAPs	Auxilin HSC70 AP1	Auxilin HSC70 AP2

### COPII-coated vesicle formation

COPII-coated vesicles transport proteins and membranes from the ER to the Golgi apparatus. The crystal structure of COPII complex has revealed the five subunits: the small GTPase Sar1p and two heterodimeric complexes, Sec23p-Sec24p [12] and Sec13p-Sec31p [13] (Fig. 1a). These proteins support vesicle budding, cargo selection, coat assembly, and uncoating of ER derived COPII-coated vesicle (Fig. 1b).

The small GTPase Sar1p acts as a “seed” in the stage of initiation of COPII-coated vesicle formation. In a microsome-based vesicle formation assay, it was found that Sar1p buried an amphipathic helix into the ER membrane [19], when it was transformed to Sar1p-GTP by mSec12, which is an ER-linked membrane protein that functions as a guanine exchange factor (GEF) for Sar1p [20]. Afterwards, Sar1p-GTP assembles the vesicle coat by sequentially recruiting the heterodimeric Sec23p-Sec24p subcomplex of COPII. Sec24p is not only involved in the assembly of the COPII coat, but also serves as a platform for cargo selection by recognizing cargo molecules with the specific regions such as diphenylalanine motif. A recent structural study of COPII suggested that Sec24p might have multiple cargo binding sites that are capable of capturing multiple cargoes, although further evidences are still needed [21]. Moreover, the membrane associated proteins of p24 family are recruited to COPII-coated vesicles by binding to Sec23p via a cytosolic diphenylalanine motif [22]. These membrane proteins, acting as cargo adaptors, are required for efficient ER-to-Golgi transport of cargo proteins [23–25]. At the same time as cargo selection, Sec23p also recruits the rod-shaped heterotetramer of Sec13p-Sec31p complex through a flexible proline-rich domain (PRD) in the C-terminal half of Sec31p [26]. Structural studies reveal that the heterotetramer of Sec13p-Sec31p complex consists of a β-propeller domain and an α-solenoid domain at N-terminal half of Sec31p, and these domains are separated by a blade insertion motif, which binds to the Sec13p β-propeller domain to form the heterotetrameric complex (Fig. 1a) [13, 26, 27]. Thereafter, with the assembly of this larger coating complex, it induces the formation of coated carriers by bending the membrane in the stage of coat assembly [13, 28–30]. Then the COPII coat complex gradually coalesces into an icosidodecahedral polymer shell, and segregates vesicle from the donor membrane [24, 31, 32]. In the stage of membrane scission, Sec23p acts as a GTPase-activating protein (GAP) to activate Sar1p-GTP hydrolysis, leading to the cleavage of vesicle membrane [33, 34]. Sar1p-GDP is thought to be released after GTP hydrolysis, resulting in vesicle uncoating before the vesicle fuses with the target membrane (Fig. 1b).

For the transport of large and irregular cargoes from the ER, such as procollagen and chylomicron, it requires the formation of COPII-coated mega-vesicles with a diameter larger than 300 nm [35]. The formation of COPII-coated mega-vesicles is similar as regular COPII-coated vesicles (Fig. 1b), except the involvement of three TANGO1 family proteins: cTAGE5, TANGO1 and short TANGO1. Structurally, the N-terminus of TANGO1 contains a SH3-like domain, which binds to collagens via HSP47, while the cytoplasmic domain of cTAGE5 contains two coiled-coil (CC) domains and a PRD that is similar to TANGO1 [36]. The immunofluorescence and co-immunoprecipitation (Co-IP) experiments revealed that the CC1 domain was essential for the recruitment of mSec12 to the ERES [37]. Moreover, TANGO1-family proteins form a ring-like structure and such a ring filament would resist to the elastic stresses and prevent membrane from further shrinking [36]. Additionally, this ring will exert a tight control over the timing and the activity of Sar1p-GTP hydrolysis at the base of a COPII bud [36]. Furthermore, TANGO1 recruits ER-Golgi intermediate compartment (ERGIC) membranes via the CC1 domain. The ERGIC membrane can fuse with cargo-enriched domain at ERES by the SNARE proteins, leading to the growth of the cargo-enriched domain into a tube-like structure. Once the tubule is large enough to accommodate these large and long cargos, the SH3-like domain and PRD of TANGO1 dissociate from cargo molecules, and the Sec23p-Sec24p subcomplex, respectively. Afterwards, the Sec13p-Sec31p subcomplex is recruited to the neck of the tubule, leading to the membrane cleavage and generation of COPII-coated mega-vesicles from the ER [35].

### COPI-coated vesicle formation

COPI-coated vesicles transport cargo proteins and lipid molecules from the Golgi membrane to the ER membrane, or between Golgi membrane compartments. The cryo-electron microscopy (cryo-EM) structure of COPI [38] revealed that the coatomer, multimeric complexes are composed of seven core subunits in the cytosol including α-COP, β’-COP, ε-COP, β-COP, δ-COP, γ-COP, and ζ-COP (Fig. 1 c), which are divided into cage-like subcomplex (B-subcomplex) and linker-like subcomplex (F-subcomplex). The B-subcomplex is a trimeric αβ’ε-COP, consisting of α-, β’-, and ε-subunits, while the F-subcomplex is a tetrameric βδγζ-COP, consisting of β-, γ-, δ-, and ζ-subunits [14–17, 39–42]. The B-subcomplex binds membrane-anchored dilysine cargo via the N-terminal WD-repeat domains of α-COP and β’-COP [43, 44], while the F-subcomplex is recruited by ADP-ribosylation factor 1 (Arf1)-GTP binding to γ-COP and β-COP subunits [45]. Coat assembly starts when the B-subcomplex, the F-subcomplex and intercalated cargoes are highly concentrated on the membrane through multivalent interactions (Fig. 1d).

The small GTPase Arf1 is activated to initiate the formation of COPI-coated vesicles by interacting with the homology downstream of Sec7d-1 (HDS1) and HDS2 domains of GBF1 (a guanine nucleotide exchange factor for Arf1) [46]. After initiation, the *cis*-Golgi localized GBF1 recruits the coatomer to the membrane as intact complexes via binding to the appendage domain of γ-COP [47]. The subunits of yeast β’-COP and part of α-COP were crystallized to form a triskelion-like structure which had been proposed to form a polygonal cage as shown in Fig. 1c [14]. In the crystal structure of the B-subcomplex, ε-COP binds to the C-terminal domain of α-COP, while β’-COP binds to a part of α-COP (Fig. 1c) [14]. Moreover, at the stage of cargo sorting, cargo molecules are selectively packaged into COPI-coated vesicles for transport. The mechanism of cargo sorting involves the interaction among the Golgi membrane, specific motif sequences in the cargo molecules, and different subunits of COPI. For example, after the recruitment of Arf1-GTP and COPI onto the *cis*-Golgi membrane, the α-solenoid domain of β’- and α-COP form an arch above the βδγζ-COP subcomplex by orienting their N-terminal β-propeller domains, such that the dilysine motif (K(X)KXX, K, Lys; X, any amino acid), or motif like RKR (R, Arg; K, Lys) of cargo binding site locates on the Golgi membrane [38, 48], and recruits molecules such as escaped ER-resident protein cargoes and soluble ER protein cargoes, which are then retro-transferred from the Golgi apparatus back to the ER [39]. When the COPI complex further concentrates and binds to cargo molecules, the B-subcomplex and the F-subcomplex intertwine to form a triply folded structure, which are connected by flexibly attached domains. One set of interactions is formed by the μ-homology domain of δ-COP and another by ε-COP and the C-terminal domain of α-COP [38]. At the stage of coat assembly, this triply folded structure deforms the planar Golgi membrane, leading to positive curvature and budding of the vesicles. In the late stage of the COPI-coated vesicle formation, the Golgi membrane is dominated by negative curvature. In morphometrical and biochemical assays, it was found that BrefeldinA-ADP-ribosylated substrate (BARS), a peripheral Golgi membrane protein, are essential for COPI-coated vesicle membrane scission [49]. In the stage of membrane scission, BARS together with COPI-associated proteins (Arf1, Arf-GAPs, and coatomer) drive the COPI complex gradually shrink to a narrow neck, and a spherical cage-like vesicles are formed via splitting the neck by phosphatidic acid that is produced by phospholipase D2 (PLD2) [50]. After the COPI-coated vesicles detaching from the Golgi membrane, Arf1-GTP is hydrolyzed under the action of Arf1-GAPs, in which Arf1-GAPs form a myristoyl-binding pocket, allowing to initiate the disassembly of the COPI complex and the uncoating of the vesicles. Last, a synthetic peptide of FFXXRRXX (F, Phe; R, Arg; X, any amino acid) with the sorting signal at the C-terminus of the p24 protein hp24a, was reported to significantly inhibit Arf1-GTP hydrolysis, while other peptides with the same sorting signal have no effect. It indicates that different cargoes may have different effects on the rate of vesicle uncoating [51], although the detailed molecule mechanism of Arf1-GTP hydrolysis and uncoating of COPI-coated vesicles is not fully understood.

### Clathrin-coated vesicle formation

CCVs transport cargo molecules from the *trans*-Golgi network (TGN) membrane to the endosome membrane, and from the plasma membrane to the endosome membrane (so called endocytosis). As observed from crystal structure (Fig. 1e), clathrin resembles a spider-like molecule with three legs radiating from a central hub [18, 52] Antiparallel interactions of the legs of triskelions centered on adjacent vertices of the lattice allows the cage to be built [53, 54]. The CCVs formation from plasma membrane and Golgi membrane shares a conserved molecule mechanism except the recruitment of different adaptor proteins and cargo molecules, in which the adaptor protein 2 (AP2) and cargos containing YXXΦ (X, any residue; Φ, a residue with a bulky hydrophobic side chain) motif, (D/E)XXXL(L/I) motif (D, Asp; E, Glu; L, Leu; I, Ile), and LL motif ((-)(2–4)XLL, (-), negatively charged residue; X, polar residue; L, Leu) are recruited to the plasma membrane by Fer/CIP4 homology domain only protein 1/2 (FCHO1/2), eps15, eps15R (eps15 related), intersectin1/2 and phosphatidylinositol-4,5-bisphosphate (PI(4,5)P2) [55–59], while AP1, Golgi-localized, gamma-adaptin ear homology, Arf-binding protein protein (GGA protein) and cargo containing (D/E)XXXL(L/I) motif and LL motif are recruited to TGN by Arf1-GTP and phosphatidylinositol-4-monophosphate (PI4P) [60, 61].

Here we take the endocytic pathway originating from the plasma membrane as an example to discuss the molecule mechanism of the CCV formation (Fig. 1f). In mammalian cells, the endocytic proteins consists at least of the adaptor proteins FCHO1/2, AP2, and the scaffold proteins eps15, eps15R, and intersectin1/2 in the initiation [54, 57, 62, 63]. The CCV formation is initiated by the increase of the endocytic proteins at the plasma membrane. FCHO1/2 locates to plasma membrane via PI(4,5)P2, and recruits eps15, eps15R, intersectin1/2 via the C-terminal AP2-μ homology domain (μHD), increasing the likelihood of initiating endocytic events [57]. Afterwards, AP2 is recruited by the μHD of FCHO1/2 and Asp-Pro-Phe triplet-based motif of eps15/eps15R [64], while clathrin is recruited by AP2 when it binds to PI(4,5)P2 and YXXΦ- and LL-containing cargoes [58, 59, 65], followed by the clathrin-coated pit (CCP) formation and the coat expansion. In the stage of cargo selection, clathrin recruits various membrane cargo proteins to the clathrin-coated site, via the adaptor proteins such as AP2, and the accessory proteins such as AP180 and epsin [7]. In the stage of coat assembly, clathrin bends the membrane during vesicle formation in the assistance of its adaptors. For example, crystal structure revealed that the amphipathic helix of the epsin N-terminal homology (ENTH) domain of epsin has createed local membrane defects and facilitates membrane curvature formation [66]. As the clathrin lattice rapidly reorganizes and propagates, membrane deformation is driven to provide the lattice flexibility to accommodate the changes in membrane curvature [67]. In the step of membrane scission, detachment of the coated vesicles from the plasma membrane is mediated by GTPase protein dynamin, which mechanically pushes out their junctions via proteolysis of GTP [68]. Other membrane binding proteins also play important roles in membrane scission, including endophilin, amphiphysin, formin-binding protein 17 (FBP17), SNX9/18, and so on [7, 67, 69–72]. Before vesicle fusion, CCVs need disassembly of the clathrin lattice during uncoating stage. Auxilin is a member of the DnaJ class of proteins that contains a conserved J domain [73] and activates the ATPase HSC70 [74]. Auxilin locates to CCVs by its clathrin binding domain, and recruits the uncoating protein HSC70 [75]. In the response of the hydrolysis ATP, HSC70 disrupts clathrin–clathrin interactions, leading to the disassembly of the clathrin coat. And then HSC70 is released and reused for the next round of vesicle formation [67].

## Intracellular vesicle transport

**Fig. 2:**
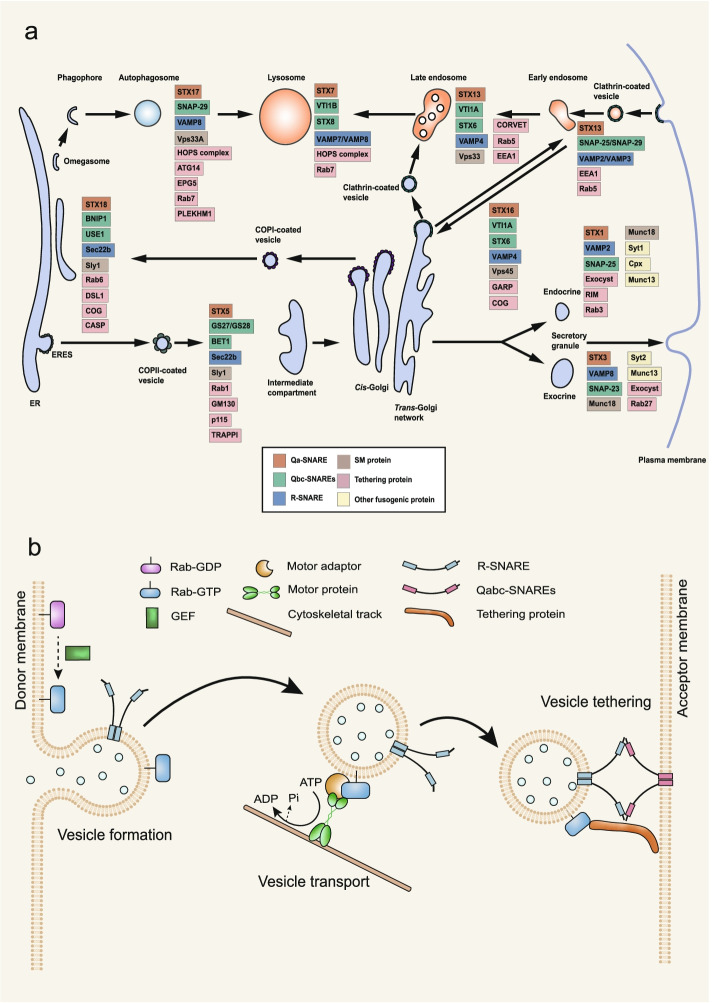
Intracellular vesicle trafficking pathways. **a** The SNAREs, SM proteins, tethering proteins and other fusogenic proteins in intracellular vesicle trafficking pathways. In mammalian cells, intracellular vesicle trafficking includes the ER-to-Golgi transport, the retrograde Golgi-to-ER transport, the TGN transport, the endocytic pathway, the membrane trafficking in autophagy and others. Different SNAREs and fusogenic proteins are assigned to different vesicle trafficking pathways. (Red, Qa-SNARE family; Light green, Qbc-SNARE family; Blue, R-SNARE family; Brown, SM protein; Pink, Tethering protein; Yellow, other fusogenic protein). Vps45, Vacuolar protein sorting 45 homolog; GARP, Golgi-associated retrograde protein. **b** The working model of vesicle transport and tethering. Rab GTPases mediates vesicle transport along the cytoskeleton tracks (actin filaments or microtubules) by indirectly binding via motor adaptors or directly binding to motor proteins. After cargo containing vesicles being loaded, motor proteins drive vesicles moving along cytoskeleton tracks to the destination by hydrolysis of ATP. Afterwards, GTP-bound Rab recruits tethering proteins to dock vesicles with the acceptor membrane. The copyright permission of panels a and b are from [3, 78]

Intracellular vesicle transport includes the ER-to-Golgi transport, the retrograde Golgi-to-ER transport, the TGN transport, the endocytic vesicle transport, and the membrane trafficking in autophagy (Fig. 2a). The intracellular vesicle transport relies on a cytoskeletal track, which involves Rab GTPases, motor adaptors, as well as motor proteins such as kinesin, dynein, myosin (Fig. 2b) [76–80]. Rab GTPases belong to the Ras superfamily of small GTPases [81]. GTP-bound Rab regulates intracellular vesicle transport by binding to the vesicle membrane and recruiting the effector molecules, such as motor proteins, motor adaptors, while GDP-bound Rab is inactive and distributed to the cytosol [81]. Each Rab protein localizes in a specific cellular compartment and recruits a different set of effector molecules, realizing precise spatial regulation of vesicle transport [81, 82]. Different proteins regulate different vesicle transport pathways by untilzing a conserved molecular mechanism in vesicle translocation, vesicle tethering and vesicle fusion with target membrane. We summarize the corresponding factors involved in the vesicle transport pathway in Table 2.

**Table 2:** Classic proteins involve in different vesicle transport pathways

Transport pathways	Rab GTPases	Motor adaptor	Motor protein	Cytoskeleton	Tethering protein
ER to Golgi transport	Rab1	Sec23	Dynein-dynactin complex, KIF5B	Microtubule	TRAPPI, p115, GM130
Retrograde transport from the Golgi to the ER	Rab6A	BicaudalD	COPI-dependent: Rabkinesin-6, KIF1C COPI-independent: Dynein-dynactin complex	COPI-dependent: Microtubule COPI-independent: Microtubule	COPI-dependent: Dsl1, COG, CASP
Transport from TGN to plasma membrane	Endocrine secretion: Rab3 Exocrine secretion: Rab27, Rab3D	Endocrine secretion: DENN/MADD Exocrine secretion: MyRIP	Endocrine secretion: KIF1A Exocrine secretion: Myosin V	Endocrine secretion: Microtubule Exocrine secretion: Actin	Endocrine secretion: Exocyst, RIM Exocrine secretion: Exocyst
Transport from TGN to endosome	Rab14	/	KIF16B	Microtubule	CORVET
Transport from endosomes to TGN	Rab6	SNX6	Dynein-dynactin complex	Microtubule	GARP, COG
Transport from plasma membrane to early endosome	Rab5	Dab2	Myosin VI	Actin	EEA1
Transport from recycling endosome to plasma membrane	Rab11	FIP2	Myosin Vb	Actin	Exocyst
Transport from endosome to late endosome	Rab5	HOOK	Dynein-dynactin complex	Microtubule	/
Transport from endosome to lysosome	Rab7	RILP	Dynein-dynactin complex	Microtubule	HOPS
Transport from autophagosome to lysosome	Rab7	FYCO1 LC3	Kinesin-1	Microtubule	EPG5, HOPS, ATG14, PLEKHM1

### ER-to-Golgi transport

Most membrane proteins, secretory proteins and lipids are synthesized in the ER, and then transported to different membrane organelles, which is critical for protein secretion and sorting [83, 84]. Newly synthesized proteins are transported to the Golgi apparatus via the ER-derived COPII-coated vesicles (Fig. 2a) [84]. As discussed in the section of vesicle formation, COPII-coated vesicles can cluster into larger vesicular-tubular structure by homotypic fusion [85]. This vesicular-tubular structure forms the ERGIC [86], an organelle before the cargos being transported to the Golgi apparatus.

Vesicle transport from the ER to Golgi is dependent on the dynein-dynactin complex, which crosslinks COPII-coated vesicle and microtubule [87, 88]. It was found that the Sec23p subunit of the COPII directly bound to the C-terminal cargo-binding domain of the p150Glued subunit of the dynein–dynactin complex by two-hybrid screening experiments [89]. In addition, the N-terminal region of p150Glued contains the cytoskeleton-associated protein glycine-rich domain, the basic domain, and the serine/proline-rich domain [90]. And it was found that the p150Glued interacted with the dynein and microtubules via its microtubule binding domain in the magic angle spinning NMR spectroscopy [91]. After cargo containing COPII-coated vesicles loaded, the dynein–dynactin complex drives COPII-coated vesicle moving along microtubule track to the Golgi apparatus by hydrolysis of ATP via the AAA (ATPases associated with diverse cellular activities) subunit of dynein [89, 92].

After COPII-coated vesicles are transported to the *cis*-Golgi network (CGN), the transport protein particle I (TRAPPI) is required for COPII-coated vesicle tethering [93]. It was found that the Bet3 subunit of TRAPPI could bind to the Sec23p subunit of COPII by the Pull-down experiments [94]. In addition, TRAPPI also acts as GEFs that specifically activate Rab1 by catalyzing GDP/GTP nucleotide exchange [94]. Rab1 then recruits its effector proteins golgins such as p115, Golgi matrix protein 130 (GM130), to tether COPII-coated vesicles to the CGN [95, 96]. Golgins are anchored to the Golgi membrane through their C-terminus. In AFM experiments, it was found that golgins could extend a considerable distance into the surrounding cytoplasm due to their coiled-coil structure property, which ensures the capture of vesicles at a long range and increases the efficiency of vesicle trafficking [97, 98].

Membrane fusion between COPII-coated vesicle and the CGN relies on a conserved SNARE complex consisting of syntaxin-5 (STX5), Golgi SNAREs of 27 kDa (GS27)/28 kDa (GS28), blocked early in transport 1 (BET1), and Sec22b [3]. Each of the SNARE proteins contributes one SNARE motif, and the formation of the four-helical bundle of SNARE complex drives membrane fusion between the COPII-coated vesicle and the Golgi membrane. The Sec1/Munc18 (SM) protein Sly1 binds to the N-terminal peptides of STX5 via its N-terminal domain, loosening the closed conformation of STX5 to facilitate the SNARE complex formation [99–101]. The detailed molecular mechanisms of SNARE and SM protein mediated vesicle fusion are discussed in section of molecular mechanism of vesicle fusion. Dysfunctions of these fusogenic proteins could cause various physiological deficits in cells. For example, it was found that the misfolded Rab1 led to the dysfunction of the ER to Golgi transport, genetically and pathologically causing neurodegeneration in amyotrophic lateral sclerosis (ALS) in mice [102]. STX5, as an integral component of the ER-derived COPII-coated vesicles, plays a role in maintaining the morphology of the Golgi apparatus [103]. GS27 and GS28 are important membrane trafficking proteins between the ER and the Golgi apparatus and between the Golgi subcompartments [104]. In the functional assays for the assessment of the pathogenicity, it was found that mutation of GS27 resulted in progressive myoclonus epilepsies [105, 106]. BET1 mainly exists at the ER and the *cis*-Golgi membrane. Downregulation of BET1 by siRNAs impairs the ER-to-Golgi transport [107]. Furthermore, the ER protein Sec22b promotes efficient membrane fusion in both anterograde and retrograde transport [108], and mutation of Sec22b has been implicated in atherosclerosis and Alzheimer's disease (AD) [109–111].

### Retrograde Golgi-to-ER transport

The retrograde Golgi-to-ER trafficking is responsible for the recovery and transport of escaped proteins or recycled membrane back to the ER [112]. This retrieval is critical for maintaining the homeostasis of the ER and the Golgi apparatus in mammalian cell [113]. The COPI-coated vesicles mediate the retrograde transport from the Golgi apparatus to the ER (Fig. 2a) [114–116].

The transport of COPI-coated vesicles from the Golgi apparatus to the ER is dependent on GTPases Rab6A and motor kinesin family proteins. Golgi located Rab6A is one of Rab6 isoforms that are ubiquitously expressed in mammalian cells. It was found that GTP-bound Rab6A was active and bound to COPI-coated vesicles via hydrophobic geranylgeranyl modified cysteine residues at C-terminus, meanwhile Rab6A also directly bound to the C-terminal domain of the motor protein Rabkinesin-6 on microtubule in two-hybrid assay and Co-IP experiments [117]. In addition, kinesin family member 1C (KIF1C), as a member of the Unc104 subfamily of kinesin-like proteins, also involved in vesicle transport from the Golgi apparatus to the ER [118]. It was reported that the C-terminus of KIF1C directly bound to Rab6A, while its N-terminal domain moved along microtubules [119]. Note that, some reports also suggested the existence of a COPI-independent Golgi-to-ER transport which is regulated by the dynein-dynactin motor complex and its adaptor BicaudalD [120]. And this COPI-independent Golgi-to-ER transport may be responsible for the retrograde transport of glycosylation enzymes and Shiga-like toxin through tubular carriers rather than vesicles [121–123].

The tethering of COPI-coated vesicles to the ER is mediated by Dsl1 [124]. The Dsl1 complex (also called STX18 complex) consists of three subunits (Dsl1, Sec39 and Tip20) [124]. The Dsl1 complex is a tethering complex that located in the ER and recognizes the Golgi-derived COPI-coated vesicles [125]. In the Pull-down experiments, it was found that COPI-coated vesicles were tethered to ER membrane by the central acidic domain of the Dsl1 interacting with the COPI subunits (α-, ɛ-, and δ-COP) [124, 126]. In addition, the conserved oligomeric Golgi (COG) complex, and Caspase (CASP) (the alternatively spliced product of the gene encoding the CCAAT-displacement protein transcription factor) also act as tethering proteins to associate COPI-coated vesicles with ER membrane [127–129].

Once vesicle tethered, membrane fusion between the COPI-coated vesicle and the ER will occur by the formation of SNARE complex consisting of STX18, B-cell lymphoma-2 interacting protein 1 (BNIP1), unconventional SNARE (primary sequence is less conserved in SNARE motif) in the ER1 (USE1), Sec22b [3]. The ER membrane-localized STX18, BNIP1, and USE1 form a ternary SNARE complex with Sec22b on vesicle to promote membrane fusion, thereby transporting the ER proteins from the ERGIC and CGN back to the ER [130]. The SM protein Sly1 also involved in mediating the membrane fusion between the COPI-coated vesicle and the ER by interacting with STX18 via its conserved N-terminal motif [99, 131]. The consequence of the membrane fusion driven by these fusogenic proteins is to maintain various normal physiological functions in cell. For example, knockdown of STX18 induces segregation of the smooth and rough ER, suggesting a role of STX18 in the organization of the ER membrane [132], while deletion of BNIP1 disrupts the three-way junctions of the ER network [133]. Mammalian USE1 is also known as MAPK-activating protein PM26 or p31, and deletion of USE1 causes death of mice due to the disruption of ER structures [134, 135].

### *Trans*-Golgi network transport

The TGN, as a cargo sorting station, generates distinct transport carriers for various destinations [136]. Membrane trafficking from the TGN is crucial for both the endocrine and exocrine cells. Secretory vesicles are formed at specific membrane region of the TGN via envelopment of the dense-core aggregates of secretory proteins by clathrin [137–139]. TGN transport includes the vesicle transport from the TGN to plasma membrane, the vesicle transport from the TGN to endosome, and the vesicle transport from endosome to the TGN (Fig. 2a). Here, we only focus on the molecular mechanism of vesicle transport from the TGN to plasma membrane which is well established and shares a conserved molecular mechanism with other TGN transport pathways (Table 2) [140–146].

In endocrine secretion, the vesicle transport from the TGN to plasma membrane involves Rab3, the adaptor protein DENN/MADD and the motor protein KIF1A. In presynapse, GTP-bound Rab3 locates on synaptic vesicles via geranylgeranyl modified cysteine residues at C-terminus and recruits its effector protein DENN/MADD, which acts as an adaptor protein to crosslink the synaptic vesicle and the CC3 domain of KIF1A [147]. The KIF1A consists of a motor domain at the N-terminal side, three CC domains and a pleckstrin homology (PH) domain at the C-terminal side. In details, the motor domain associates with microtubule via the microtubule-binding domains, while CC1 domain regulates the motor activity by inhibition of the motor domain. Moreover, both the CC1 and CC2 domain regulate motor dimerization [148, 149], and the PH domain mediates the recruitment of cargo containing vesicle via binding to PI(4,5)P2 [150]. By overexpression or knockdown of KIF1A, it was found that the overactivation of KIF1A increased the number of synaptic vesicles at active zone of the presynapse, while the depletion of KIF1A resulted in a decrease in the number of synaptic vesicles [151], suggesting that the KIF1A is important for the axonal transport of synaptic vesicles to plasma membrane [150].

The exocyst complex is required for vesicle tethering to the plasma membrane [152]. The vesicle located Rab GTPase Sec4 (the yeast homolog of Rab8), recruits the exocyst complex to secretory vesicles through interacting with the subunit of Sec15 [153]. The exocyst consists of the eight subunits EXOC1-8 in mammalian cell, corresponding to Sec3, Sec5, Sec6, Sec8, Sec10, Sec15, Exo70 and Exo8 in yeast [152]. In the co-sedimentation experiments, it was found that EXOC1 bound with PI(4,5)P2 on plasma membrane via its PH domain-like region at its N-terminus [154], while EXOC7 bound with PI(4,5)P2 via a patch of basic residues at its C-terminus [155]. In mammalian cell, Rab-interacting molecule (RIM), as the effector protein of Rab3, is also required for vesicle tethering to the plasma membrane. In liposome binding experiments, it was found that RIM could interact with vesicle located GTP-bound Rab3 via its two α-helical regions at N-terminus, and could bind to PI(4,5)P2-containing membrane via its C2B domain [156]. In addition, it was repored that RIM protein also involved in tethering N- and P/Q-type Ca^2+^ channels to presynaptic active zone via a direct PDZ-domain interaction in RIM conditional knockout mice [157]. Moreover, the RIM’s Zinc finger (ZF) domain could interact with C2A domain of Munc13 to facilitate vesicle tethering [158].

CCVs transport from the Golgi to endosomes, lysosomes, or plasma membrane, also relies on SNARE proteins [3]. For example, neurotransmitter release is mediated by a ternary SNARE complex consisting of STX1A, synaptosomal-associated protein 25 kDa A (SNAP-25A), and vesicle associated membrane protein 2 (VAMP2) [159]. A ternary SNARE complex consisting of STX7, vesicle transport through interaction with t-SNAREs 1B (VTI1B), STX8 and VAMP8 drives homotypic fusion of late endosomes [160, 161], while VAMP7 determines heterotypic fusion of late endosomes [162]. In addition, cargo molecules from endosomes can also be transported back to the TGN. The ternary SNARE complex involved in the endosome-to-TGN trafficking is STX6, STX16, vesicle transport through interaction with t-SNAREs homolog 1A (VTI1A), VAMP4 [145, 163–165]. The detailed molecular mechanisms of SNARE mediated vesicle fusion are discussed in section of molecular mechanism of vesicle fusion.

### Endocytic pathway

The endocytic transport is critical for the uptake of extracellular components, receptor internalization and the regulation of cell signaling [166]. In the endocytic pathway, CCVs with internalized molecules are transported to early endosomes for cargo sorting. Some molecules such as recycling receptors and lipid membrane are transported back to plasma membrane via recycling endosomes, while others including ubiquitylated proteins and downregulated receptors are transported to late endosomes and lysosomes for degradation [167]. Endocytic vesicle transport among several intracellular organelles of early endosomes, late endosomes and lysosomes also shares a similar molecular mechanism, although the regulatory proteins are different (Fig. 2a) (Table 2) [168–174]. Here, we take the endocytic transport of the clathrin-coated endocytic vesicle from plasma membrane to endosome for example to elucidate the molecular mechanism of the key regulators involved in the pathway.

The transport of the clathrin-coated endocytic vesicle from plasma membrane to early endosomes involves AP-2, Disabled 2 (Dab2) and myosin VI [175]. The CCVs located AP-2 binds to the DPF motifs of adaptor protein Dab2 at the central region [176]. Moreover, the C-terminal region of Dab2 binds to the divergent tail domain of myosin VI, and induces the dimerization of myosin VI, which is essential for its motion activity [176]. The highly conserved motor domain of myosin VI is responsible for binding to F-actin and transporting vesicle along actin filament by converting energy from ATP hydrolysis into directional motion [176]. For more details about the endocytic transport of CCVs, we refer to the other reviews [7, 67, 166, 177].

The tethering protein early endosomal antigen 1 (EEA1) is required for the clathrin-coated endocytic vesicle tethering with early endosomes [178, 179]. EEA1 is predominantly localized to the early endosomes as the specific biomarker. The crystal structure of EEA1 revealed that EEA1 could form into a ~ 200 nm long coiled-coil homodimer, composed of an N-terminal C_2_H_2_ ZF domain and a C-terminal FYVE (Fab 1, YOTB, Vac 1, and EEA1) domain, in which the FYVE domain interacts with phosphatidylinositol-3-phosphate (PI3P), which is essential for early endosomal membrane to recruit EEA1, while the C_2_H_2_ ZF domain binds to GTP-bound Rab5 on the clathrin-coated endocytic vesicles [180]. Moreover, the tethering protein class C core vacuole/endosome tethering (CORVET) is involved in the homotypic fusion of early endosomes. Early endosomes located CORVET, consists of Vps3, Vps8, Vps11, Vps16, Vps18, and Vps33 subunit [181], in which the subcomplex of Vps3 and Vps8 physically and genetically interact with the Rab5 for early endosomes tethering [182].

A ternary SNARE complex consisting of STX13, SNAP-25/SNAP-29 and VAMP2/VAMP3 drives the heteromorphic fusion of the clathrin-coated endocytic vesicle and early endosome [10]. STX13 and SNAP-25/SNAP-29 contributes Qa- and Qbc-SNARE motifs, respectively, while VAMP2/VAMP3 contributes the other arginine-containing SNAREs (R-SNARE) motif, thereby assembling into the four-helical bundle of SNARE complex, and mediating the membrane fusion [10]. On the other hand, homotypic membrane fusion of early endosomes is essential for the formation of sorting endosomes. Homotypic membrane fusion of early endosomes relies on a conserved SNARE complex consisting of STX13, VTI1A, STX6, and VAMP4, although such SNARE pairing does not suffice to determine the specificity of early endosome fusion [183]. Besides, the SM proteins Vps33, one subunit of the CORVET/ homotypic fusion and protein sorting (HOPS), is also critical for the homotypic membrane fusion of early endosomes. In reconstituted liposome fusion assay, it was found that Vps33 promoted fusion pore opening by enhancing the formation of SNARE complex [184].

### Membrane trafficking in autophagy

Autophagy is a highly regulated catabolic process in eukaryotic cells, which uses lysosomes to degrade large protein aggregates, damaged organelles and other components to cope with internal and external stress and to maintain cell homeostasis [185]. The process of macroautophagy includes five stages: autophagy induction, nucleation process, extension of autophagosome, fusion of autophagosome and lysosome, and degradation within autophagosome [186].

The retrograde transport of autophagosomes to lysosomes involves Rab7, LC3, FYVE and coiled-coil domain-containing protein 1 (FYCO1), kinesin-1. Rab7 recruits its effector protein FYCO1, which binds to microtubule-associated proteins 1A/1B light chain 3B (LC3B) and PI3P on autophagosomes via FYVE domain [187, 188]. In immunoprecipitations and immunoblots experiments, it was found that the phosphorylation of LC3B would reduce the binding affinity with FYCO1 [189]. Moreover, bead capture based Pull-down assays indicated that the middle part of FYCO1 (residues 585–1233) bound directly to the kinesin light chain 2 of kinesin-1 [190], and protrudin, an ER protein, promotes such interaction in human and rat cell lines, which is required for the retrograde transport of autophagosomes to lysosomes along kinesin-1 associated microtubule [187, 189, 190].

Ectopic P granules protein 5 homolog (EPG5), as an effector protein of Rab7, serves as a tethering factor to ensure the specificity of autophagosome-lysosome/late endosome fusion. EPG5 is recruited to the lysosome or late endosome by interacting with ATG8 homolog human LC3B/LC3C on autophagosome via its two LC3-interacting regions with a conserved sequence (W/F/Y-X1-X2-I/L/V) [191]. Pleckstrin homology domain-containing family member 1 (PLEKHM1), as another Rab7 effector protein, is localized on late endosomes and lysosomes. It was found that PLEKHM1 bound to ATG8 family proteins, preferentially to GABARAPs, to capture autophagosome by a yeast two-hybrid system [192, 193]. ATG14, an essential autophagy-specific regulator of the class III phosphatidylinositol 3-kinase complex, promotes membrane tethering of autophagosome. In a reconstituted liposome fusion assay, it was found that ATG14 homo-oligomerization enhanced their ability to promote membrane tethering [194]. Moreover, ATG14 interacts with the SNARE core domain of STX17 through its CC domain and stabilizes the STX17-SNAP-29 complex to promote membrane fusion of autophagosomes and lysosomes [194]. The HOPS complex also plays a tethering role in autophagy. It was found that HOPS was recruited to the autophagosome membrane by binding to Rab7 and phospholipids such as PI3P in protein-lipid overlay assays [195]. In summary, multiple tethering factors may act coordinately to ensure the efficiency and specificity of vesicle tethering in autophagy.

SNAREs (STX17, SNAP-29, and VAMP8) are essential for autophagosome formation and degradation [194, 196]. STX17 is widely expressed in a variety of tissues and mainly located on the ER membrane, mitochondria and cytoplasm [197]. When autophagy is activated, STX17 and SNAP-29 are recruited to the autophagosome membrane, and interact with VAMP8 on the lysosome to form the ternary SNARE complex, which drives the membrane fusion of autophagosome and lysosome, leading to the completion of autophagy [194, 198]. Besides of SNARE protein, the SM protein Vps33A [199] also binds to the SNARE complex at the groove of the four-α-helical bundle, promoting the vesicle fusion by stabilizing the STX17-SNAP-29-VAMP8 complex [200].

## Molecular mechanism of vesicle fusion

Vesicle fusion with a target membrane is the end of a particular vesicle trafficking pathway. Vesicle fusion involves several steps, such as vesicle tethering or docking, vesicle priming, hemifusion, fusion pore opening and the SNARE complex disassembly [3, 201–203]. To achieve this, the repulsive force generated by the negatively charged lipid bilayers and the dehydration of the water layers at the lipid headgroups have to be overcome. The SNARE complex formation can produce the energy to drive two membranes fusion via the formation of four-helix bundles [204]. Without regulators, membrane fusion occurs spontaneously by a thermodynamically-driven process of the SNARE complex formation [205]. To precisely regulate the vesicle fusion, other fusogenic proteins, such as the tethering factor Rab and RIM, Ca^2+^ sensor protein Syts, Cpx, Munc18, Munc13, NSF and α-SNAP cooperate to ensure vesicle fusion event occurs at defined fusion site and under precise control [159, 202, 206, 207]. Intensive research that began over 40 years has resulted in an understanding of the molecular mechanism of SNARE-mediated vesicle fusion.

### SNARE proteins

**Fig. 3:**
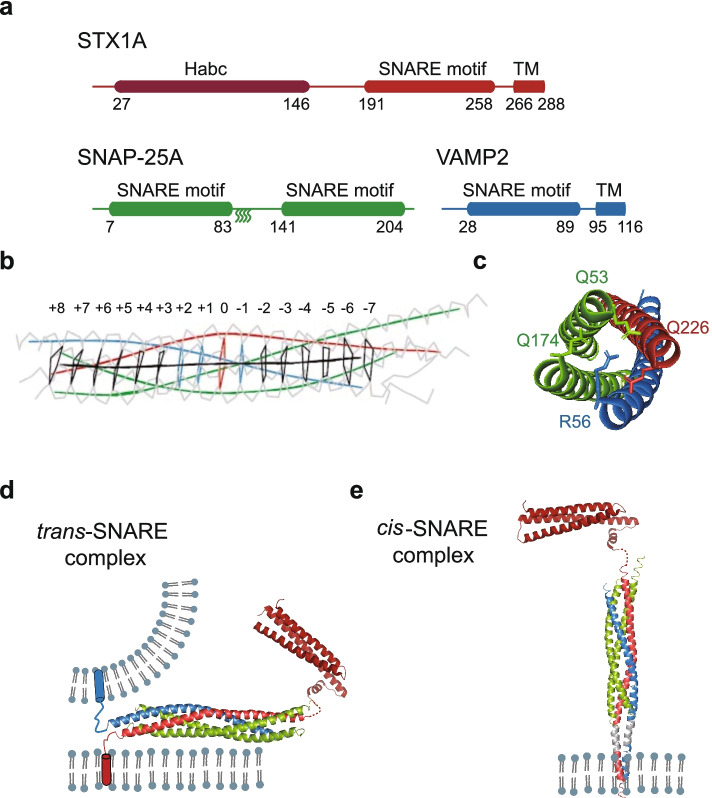
SNARE proteins and SNARE complex. **a** Domain diagrams of the STX1A (red) with Habc domain (dark red), SNAP-25A (green) and VAMP2 (blue). For SNAP-25A, the palmitoylation sites within the cysteine cluster (C85, C88, C90, C92) of the linker region are presented as curve lines. The numbers of residues are indicated below each diagram. The color code for each SNARE protein is the same in other figures. TM, transmembrane. **b** 16 stacked layers (Red: 0; Blue: -1, + 1 and + 2, that are closest to the ideal leucine-zipper geometry; Black: others) of the SNARE complex are indicated in Cα traces (gray), the superhelical axis (black), the helical axes of STX1A, SNAP-25A and VAMP2 are shown as red, green, and blue respectively. **c** The side chains involved in the ionic “0” layer of SNARE complex are shown as sticks. **d** The ribbon diagram shows the structure of the *trans*-SNARE complex in *Rattus norvegicus* (PDB ID: 1SFC) [217] with STX1A Habc domain (PDB ID: 3C98) [218] on two opposing membranes. **e** The ribbon diagram shows the structure of the *cis*-SNARE complex in *Rattus norvegicus* (PDB ID: 3IPD) [219] on plasma membrane after a full fusion event. The copyright permission of panels b and c are from [217]

SNARE proteins involve in most of the intracellular vesicle trafficking pathway except the homotypic fusion between the mitochondria and the ER membrane [208]. The nomenclature of SNARE proteins stems to their discovery as membrane receptors for soluble NSF attachment protein (SNAP) [209–215]. SNARE proteins have a superfamily of 36 homologs in human, here we mainly focus on the best characterized neuronal SNAREs. SNARE proteins are characterized by the SNARE motif in the membrane proximal region, which is evolutionary conserved domain of 60 ~ 70 residues with heptad repeats (Fig. 3a). The SNARE hypothesis was proposed in 1993, in which it postulated that a distinct vesicle-SNARE (v-SNARE) pairs with the target membrane-SNARE (t-SNARE), and such specific interaction drives two membranes to fuse [216].

The first identified SNAREs in mammalian cells were synaptic STX1A [209], SNAP-25A [213] and VAMP [214], while the yeast SNAREs were independently discovered via genetic screening [212]. STX1A and SNAP-25A are generally located on the plasma membrane, while VAMP2 is primarily located on the synaptic vesicles. Both STX and VAMP are anchored to membrane via a single helical transmembrane domain at the C-terminus, whereas SNAP-25A resides on the plasma membrane via the palmitoylation modification of the four cysteine residues on the linker region (Fig. 3a). In the ternary neuronal SNARE complex, the four SNARE motifs form a parallel four-helix bundle, of which SNAP-25A contributes two SNARE motifs, STX1A and VAMP2 each contribute one SNARE motif, respectively [217].

The SNARE complex of four-helix bundle consists of 16 stacked layers of interacting side chains (Fig. 3b) [220]. The “-1”, “ + 1” and “ + 2” layers of the complex are at the center position, which are closest to the ideal leucine-zipper geometry and amino acid composition (Fig. 3b). In the middle of the SNARE motif, it is a highly conserved “0” layer (Fig. 3b), composed of one arginine residue from VAMP2, one glutamine residue from STX1A, and two glutamine residues from SNAP-25A (Fig. 3c). According to this remarkable feature, SNARE proteins are also classified as glutamine-containing SNAREs (Q-SNARE) and R-SNARE, respectively. The QabcR-complex groups in “0” layer of the SNARE complex are highly conserved throughout all species [220].

SNARE proteins that are located in opposing membranes form the *trans*-SNARE conformation (Fig. 3d), and drive membrane fusion by releasing free energy (~ 36 k_B_T) [204, 221] during the zippering of the SNARE complex, which ends in the *cis*-SNARE conformation (Fig. 3e) [219]. The fusion pore is a vital transient state in the final step of each trafficking pathway. The process of fusion pore opening and dilation is highly dynamic, which might determine the fate of trafficking vesicles, either fusing with the plasma membrane or via a “kiss-and-run” route (fusion pore closes rapidly without full dilation) [222]. The copy number and dynamics of *trans*-SNARE complex are critical for fusion pore formation [223]. To induce lipid stalk formation, the energy required to overcome the hydration-force barrier is around 40 ~ 90 k_B_T [224]. Considering the additional energy needed for the pore formation and dilation, more energy would be required to drive a full fusion event. Thus, one SNARE complex may be sufficient for lipid exchange between two membranes, i.e. a hemifusion intermediate state [225], but more SNARE complexes are required for a full fusion event [226–228].

Post-translational modifications (PTM) of SNARE proteins, for example phosphorylation, palmitoylation, acetylation, and O-GlcNAcylation, play an important role in the regulation of membrane trafficking [229]. In the vesicle trafficking pathway of autophagy, deacetylation of STX17 enhances the binding to SNAP-29 and facilitates the formation of the STX17-SNAP-29-VAMP8 complex, thereby further promoting autophagosome-lysosome fusion [198]. Additionally, both O-GlcNAcylation of SNAP-29 and phosphorylation of VAMP8 hinder the SNARE complex assembly [230–233]. Moreover, PTM of SNARE proteins are also involved in other vesicle trafficking pathways. Phosphorylation of the residues within the SNARE motif of VAMP8 inhibits mast cell secretion [232], which might be important for preventing overreach reaction such as anaphylactic shock. Palmitoylation of SNAP-25A is essential for exocytosis in neuroendocrine cells [234], while ubiquitination of STX5 inhibits the SNARE complex assembly and disrupts Golgi membrane fusion during early mitosis [235]. Taken together, PTM of SNAREs is crucial to their localization and the SNARE complex formation in membrane trafficking pathway.

### Synaptotagmin

**Fig. 4:**
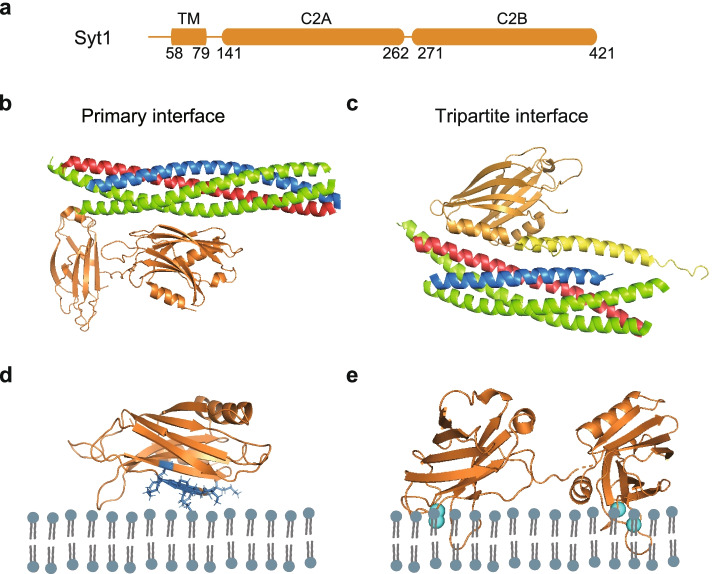
Syt1 and its interactions with SNARE complex and membrane. **a** Domain diagram of Syt1. Residue numbers are indicated below the diagram. **b** Crystal structure of the primary interface of SNARE-Syt1 complex in *Rattus norvegicus* (PDB ID: 5CCI) [240]. **c** Crystal structure of the tripartite interface of SNARE-Syt1-Cpx1 complex in *Rattus norvegicus* (PDB ID: 5W5D) [241]. **d** Model of polybasic region of C2B domain interacting with the anionic membrane (PDB ID: 1K5W) [242]. The side chains of the residues in the polybasic region (K313, K321, R322, K324, K326, K325 and K327) are shown in blue sticks. **e** Crystal structure of the tandem Syt1 C2AB (orange) domains in *Rattus norvegicus* (PDB ID: 5KJ7) [239] in the presence of Ca^2+^ (cyan sphere)

The Syts, as the Ca^2+^ sensor protein, play a key role in regulating Ca^2+^-triggered membrane fusion [236, 237]. Syts are an evolutionary conserved family of proteins that consist of N-terminal single transmembrane domain, an unstructured linker region, and two cytoplasmic protein Kinase C-like C2 domains, termed C2A and C2B, respectively (Fig. 4a) [236, 238, 239].

There are 16 isoforms of mammalian Syts [243]. Among these isoforms, Syt1, Syt2 and Syt9 are the Ca^2+^ sensor for evoked synchronous synaptic vesicle fusion or secretory granule secretion. In addition, Syt7 acts as a redundant Ca^2+^ sensor for Ca^2+^-dependent asynchronized release [244–246]. Here, we only focus on Syt1 isoform that is located on the synaptic vesicle and is critical for synchronous Ca^2+^-triggered synaptic vesicle fusion.

In absence of Ca^2+^, Syt1 binds to both the anionic membranes via the polybasic region and possibly arginine apex, as well as the SNARE complex via the primary interface and tripartite interfaces (Fig. 4b-d) [240–242, 247], which is vital for vesicle docking and priming [240, 248–259]. Note that, the interaction between the SNARE complex and the polybasic region of Syt1 was also observed in the absence of membrane environment by NMR experiments [249]. In vitro reconstitution fusion assay and cryo-EM of cultured neuron experiments demonstrated that Syt1 bound to the heterodimer of STX1A-SNAP-25A subcomplex to mediate vesicle docking [260, 261]. It was found that Syt1 clamped the frequency of miniature spontaneous fusion events in the absence of Ca^2+^ by in vitro reconstitution assay and neuronal culture experiments [262–265]. In addition, ring-like oligomers of Syt1 was observed on synthetic membrane in cryo-EM experiments, and destruction of the oligomer ring will lead to the increase of spontaneous fusion in the absence of Ca^2+^ [263, 264, 266]. However, such ring-like structure have not yet been found in cryo-ET studies of synaptosomes [267]. In another model, Syt1 and Cpx1 can lock two membranes in the pre-fusion state by interacting with the SNARE complex via a tripartite interface and a primary interface (Fig. 4b-c), which is thought to be critical in promoting Ca^2+^-triggered neurotransmitter release [241]. A different model has been recently proposed that Syt1 may dissociate from the primary complex upon Ca^2+^ binding to PI(4,5)P2 containing membrane environment, and subsequent followed by conformational change of Cpx [268]. Note that, the primary interface has been confirmed in several studies in solution as well as in neurons, while further studies are required to fully corroborate the physiological importance of the tripartite interface.

When Syt1 binds to Ca^2+^ via the region located on the bottom of the each C2 domain (Fig. 4e), synchronized neurotransmitter release occurs [269–273]. In Syt1-deficient mammalian neurons, Syt1 mutants that disrupt the Ca^2+^ binding sites of the Syt1 C2A domain can partially rescue the phenotype in the Ca^2+^-evoked neurotransmitter release, while mutants that disrupt the Ca^2+^ binding sites of the Syt1 C2B domain cannot, suggesting a more vital role of C2B domain than C2A domain [265, 274, 275]. Reducing the Ca^2+^ binding affinity of Syt1 in mice causes a correspondingly reduction in Ca^2+^ sensitivity of fusion [253, 276], proving that Syt1 is the Ca^2+^ sensor in synaptic vesicle fusion. One of established working model for Ca^2+^-triggered synaptic vesicle fusion is a synergistic interaction among Syt1, the SNARE complex, and anionic membrane. In this model, the primary interface between Syt1 and the SNARE complex [240] and the interaction between the polybasic region of Syt1 and anionic lipids [249, 268, 277] may serve as a scaffold, while the fusion loop of C2 domain inserts into anionic membrane upon Ca^2+^ binding, and produces the local positive membrane curvature on the plasma membrane, which substantially increases the fusion probability by lowering the energy barrier of hydration force [268, 278–281]. It has also been shown that Syt1-Ca^2+^ substantially promotes the fusion pores opening and expansion by cooperating with the SNARE proteins [262, 282] or interacting with membrane by two conserved arginine residues in Syt1 C2B domain [247].

Although the interaction between the polybasic region of Syt1 and the SNARE complex was observed in numerous biochemical studies and NMR experiments [249, 268], in vitro reconstitution experiments suggested that Syt1 promoted Ca^2+^-triggered vesicle fusion by binding to PI(4,5)P2-containing membrane rather than SNARE at physiological ion concentration [283]. Such controversial results under different experimental conditions suggest a rather dynamic interaction between Syt1 and the SNARE complex.

Besides, the cryo-ET analysis of mouse hippocampal synapses revealed that Syt1 loosely docked synaptic vesicles to plasma membrane within 2 ~ 12 nm by interacting with PI(4,5)P2, while the SNARE complex brought synaptic vesicles closer within 0 ~ 2 nm [284, 285]. Additionally, by functional reconstitution in liposome fusion, it suggested C2 domains were capable of decreasing the gap between synaptic vesicle and target membrane by crosslinking opposing membranes [286, 287]. Using reconstituted proteoliposome, it was found that under low ionic, Syt1 functions as a distance regulator that tethers the liposomes close enough for membrane fusion in the presence of Ca^2+^ [287]. Moreover, both in vitro and in vivo studies showed that the linker between the C2 domains, and the juxta membrane linker between the transmembrane domain and the C2A domain are also important for Syt1 function in Ca^2+^-triggered vesicle fusion, possibly by regulating the distance between two membranes [288–291].

### Complexin

Cpx, also known as synaphin, is a small cytoplasmic protein, which is largely unstructured in solution [292]. There are four homologs in the mammalian Cpx family. Cpx1 and Cpx2 mainly exist in synapse, while Cpx3 and Cpx4 are found in the optic nerve [293, 294]. The difference in subtype distribution implies the functional difference among the isomers of Cpx. Here, we focus on the homolog Cpx1 whose primary sequence is highly conserved in different species [293, 295, 296].

**Fig. 5:**
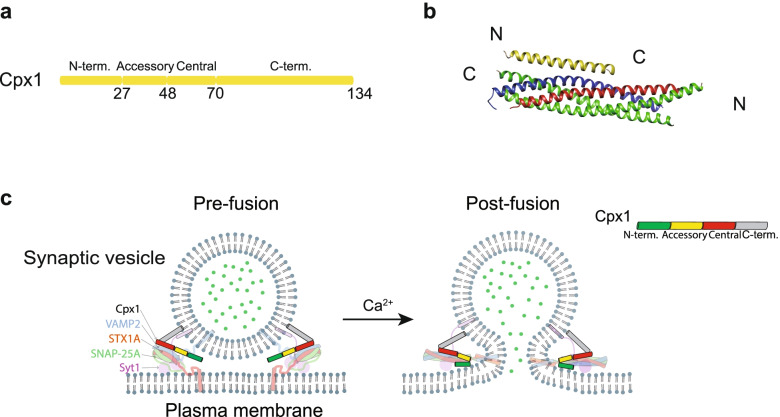
Cpx1 and its regulatory function. **a** Domain diagram of Cpx1. Residue numbers are indicated below the diagram. **b** Crystal structure of the SNARE-Cpx1 complex in *Rattus norvegicus* (PDB ID: 1KIL) [305]. **c** Models of how Cpx1 regulates synaptic vesicle fusion. In absence of Ca^2+^, the core fusion machinery is locked to a pre-fusion stage via several interactions from Cpx1: the central α-helical domain interacts with SNARE complex; the accessory α-helical domain involves in a tripartite interface with Syt1 and SNARE complex; C-terminal domain is associated with synaptic vesicle (left). When Ca^2+^ influx, Cpx1 inhibition could be released by a conformational change of the tripartite interface upon Ca^2+^ binding to Syt1, meanwhile Cpx1 synchronizes synaptic vesicle fusion via its N-terminal domain binds to C-terminus of *trans*-SNARE complex and plasma membrane (right)

Cpx1 consists of a N-terminal domain, an accessory α-helix domain, a central α-helix domain and a flexible C-terminal domain (Fig. 5a). The N-terminal domain of Cpx1 plays a key role in activation of fast synchronous release in mammalian cell [297, 298], probably via interaction with the plasma membrane and the C-terminal end of the SNARE complex [299, 300]. The accessory domain plays a role in reducing spontaneous release in cultured neuron based electrophysiology experiments and in vitro reconstituted systems, although its molecular mechanism is still controversial [301–303]. Cpx1 binds to the SNARE complex via its central α-helix domain, which inserts into a groove formed by VAMP2 and STX1A in an anti-parallel orientation (Fig. 5b) [302, 304, 305] Moreover, the C-terminus of Cpx1 is attached to synaptic vesicle via sensing the membrane curvature of synaptic vesicle, and such localization on synaptic vesicle is critical for inhibiting spontaneous release [299, 306]. Furthermore, the C-terminal domain has an effect on synaptic vesicle priming in neurons [299, 307, 308]. The C-terminal deletion of Cpx1 cannot inhibit spontaneous release in neuron, but it still stimulates Ca^2+^-triggered release in both cultured neuron and reconstitution system [299, 307–309].

Cpx1 acts both as an activator and as an inhibitor for neurotransmitter release, giving rise to a major controversy (Fig. 5c) [295]. In the presence of Ca^2+^, Cpx1 activates synchronous neurotransmitter release via its N-terminal region and the central α-helix domain binding to the SNARE complex (Fig. 5c) [292, 295, 296, 298, 303, 304, 308–313], while in the absence of Ca^2+^, Cpx1 has an inhibitory function on spontaneous release via the accessory α-helix domain and the C-terminal domain, although this function is less conserved among species (Fig. 5c) [299, 304, 308, 309, 314, 315]. Site-directed mutagenesis in *Drosophila* showed that the Cpx clamping function was predominantly maintained by its accessory helix, and molecular modeling results suggested that the Cpx accessory domain interacted with the truncated C-terminus of VAMP2 and the lipid bilayer of synaptic vesicle to prevent the SNARE complex fully assembly [316]. Energetic measurements by surface forces apparatus revealed that Cpx1 facilitated SNAREpin assembly sequentially by doubling the distance of intermembranes at which the SNAREs alone that can engage, and then clamped *trans*-SNAREpin formation by binding to the C-terminus of SNARE complex, into a half-zippered intermediate state [317, 318]. Besides, the crystal structure of SNARE-Cpx1 complex revealed that Cpx1 could organize SNAREs into zig-zag topology to prevent spontaneous fusion, although a C-terminal SNARE motif truncated VAMP2 was used to mimic *trans-*SNARE state [319]. However, the follow-up studies suggested that Cpx mutants in *Drosophila* which disrupted the zig-zag topology of SNARE-Cpx1 complex, could still rescue Ca^2+^-evoked neurotransmitter release, indicating that crosslinked the *trans*–SNARE complex by Cpx is not a prerequisite for synchronized neurotransmitter release [320]. Last, the crystal structure of the primed pre-fusion SNARE-Cpx-Syt1 complex revealed an unexpected tripartite interface and the mutations of the interface severely impaired Ca^2+^-evoked synchronous release in neurons, suggesting that interface is essential for the primed pre-fusion state [241].

### Munc13 and Munc18

**Fig. 6:**
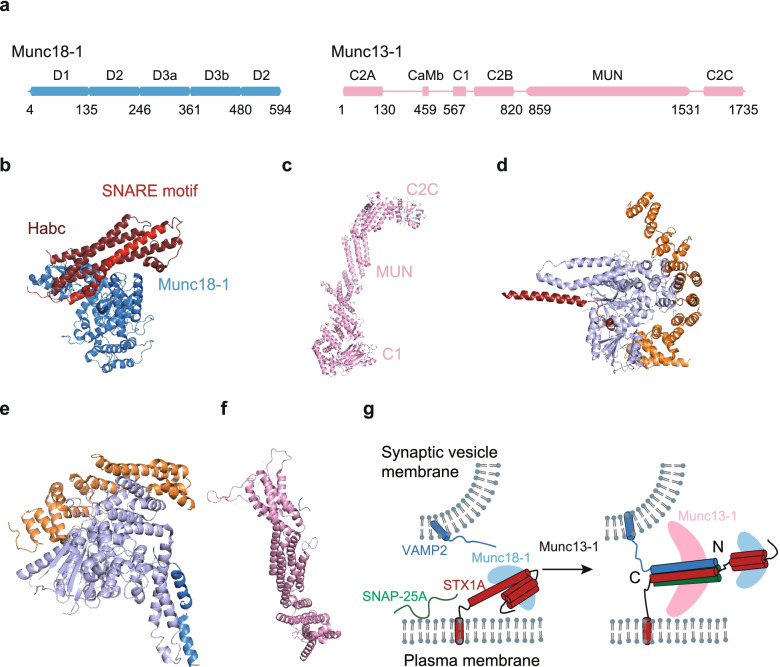
Munc18-1 and Munc13-1. **a** Domain diagrams of Munc18-1 (blue) and Munc13-1 (pink). Residue numbers are indicated below the diagrams. **b** Crystal structure of the STX1A-Munc18-1 complex in *Rattus norvegicus* (PDB ID: 3C98) [218]. The Habc domain (dark red) of STX1A locks the SNARE motifs (red) in the closed conformation by the interaction of Munc18-1. **c** Cryo-EM crystal structure of the C1C2BMUNC2C fragment of Munc13-1 in *Rattus norvegicus* (PDB ID: 7T7V) [334]. **d** Crystal structure of the Qa-SNARE Vam3 (red) bound to the SM protein Vps33 (light blue) in *Chaetomium thermophilum* (PDB ID: 5BUZ) [321]. **e** Crystal structure of the R-SNARE Nyv1 (marine) bound to the SM protein Vps33 in *Chaetomium thermophilum* (PDB ID: 5BV0) [321]. **f** Crystal structure of the VAMP2 (marine, residues 87–92)-MUN (pink) complex in *Rattus norvegicus*. (PDB ID: 6A30) [333]. **g** Model of Munc13-1 (pink) catalyzing the transfer of STX1A from the Munc18-1 (light blue)-STX1A complex to the properly assembled ternary *trans-*SNARE complex. The color codes for STX1A, SNAP-25A and VAMP2, are red, green, and blue, respectively

Munc13 and Munc18 regulate synaptic vesicle priming via orchestrating the SNARE complex properly assembly [321–325]. Here we focus on Munc13-1 and Munc18-1 that are well studied in synapse. The structure of Munc18-1 protein includes five domains (Fig. 6a) [326, 327]. Structural studies by crystallography and electron paramagnetic resonance spectroscopy revealed that Munc18-1 captured STX1A, locking it into a heterodimeric complex in a closed conformation [218, 328–330]. This state kinetically prevents the formation of ternary SNARE complex [218, 330, 331] until the presence of Munc13-1. The crystal structure of the Munc18-1-STX1A complex revealed the interaction of STX1A with the pocket formed by domains 1 and 3 of Munc18-1 (Fig. 6b) [329, 330] explaining the inability of the SNARE motif of STX1A to interact with SNAP-25A and VAMP2. Although such closed conformation is not valuable for vesicle fusion due to its hindrance to the SNARE complex formation, it may be required for the recruitment of Munc18-1 to fusion sites of active zone [332], and it may serve as template for the SNARE complex assembly [323, 333].

Munc13 enables STX1A to transit into open conformation and it acts as a chaperons to promote ternary neuronal SNARE complex formation [335–337]. Munc13-1 consists of a C1 domain, a calmodulin-binding domain, two C2 domains (C2A, C2B), a MUN domain, and another C2 domain (C2C) (Fig. 6a). The crystal structure of the C1C2BMUNC2C fragment of Munc13-1 revealed intramolecular interactions among the C1, C2B, and MUN domains (Fig. 6c) [334, 338] The C1 and C2B domain of Munc13-1 binds to diacyglycerol and PI(4,5)P2 on the plasma membrane, respectively, and regulates the priming of synaptic vesicles, probably via increasing the local concentration of MUN domain [323, 339]. The C2A domain of Munc13-1 interacts with RIM [340], which might be essential for the vesicle tethering and priming [341]. The MUN domain of Munc13 performs two functions: (1) catalyzes the transfer of STX1A from the STX1A-Munc18 complex to the ternary *trans*-SNARE complex [333, 335, 342], and (2) promotes the parallel configuration assembly of the SNARE complex with Munc18 [323, 343, 344]. Last, the C2C domain of Munc13-1 activates Ca^2+^-evoked release in chromaffin granule secretion and neurotransmission in *C. elegans*, likely via bridging two opposing membranes [345–347].

It was found a novel autoinhibitory role for the C2B domain of Munc13 by deletion of C2B of *C. elegans* Munc13 ortholog UNC-13 in electrophysiology experiments and the autoinhibition by C2B was relieved by Ca^2+^ binding to C2B and stabilized by the neighboring C1 domain [348, 349]. In addition, NMR experiments revealed the interaction between Munc18-1 and VAMP2, and this interaction was inhibited by a L348R mutation in Munc18-1 but stimulated by the D326K, which might contribute to the autoinhibition of VAMP2 [350]. Moreover, the mutation of Munc18-1 Q301D inhibited lipid mixing in a reconstituted fusion assay and its expression in Munc18-1 deficient neurons severely reduced synaptic transmission [351]. The mutations of VAMP2 that impair the function of Munc18-1 to promote *trans*-SNARE zippering, strongly inhibit spontaneous and synchronized neurotransmitter release in cultured neurons [352]. Furthermore, the crystal structures of Vps33 with Qa-SNARE Vam3 or R-SNARE Nyv1 and Munc13 with short VAMP2 fragment suggested Munc18 and Munc13 could bind to individual SNARE and align them into a correct orientation (Fig. 6d-f) [321, 323, 333]. Last, the recent functional study suggested that Munc13 promoted the proper SNARE complex assembly together with Munc18, which was critical for the physiological functions of Munc13 in priming and short term presynaptic plasticity [323]. Taken together, a working model has been proposed for Munc13 and Munc18, in which Munc13 and Munc18 may act as a template for the SNARE complex properly assembly to ensure synaptic vesicle priming (Fig. 6g).

### Cooperation of the fusogenic proteins

Intensive studies of the molecular mechanism of SNARE-mediated vesicle fusion has resulted in a possible working model. 1) As a starting complex, Munc18 locks STX into a closed conformation on the plasma membrane [353]. Secretory vesicles are recruited to a delicate fusion site by the interaction with tethering factors such as Rab, RIM and Munc13 [354–357]. 2) In the step of vesicle priming, also known as the step of ready for fusion, or readily releasable pool, Munc13 catalyzes the transit of STX from the closed conformation of Munc18-STX complex into the proper *trans*-SNARE complex [323, 342, 353, 358]. Meanwhile, via interacting with the *trans*-SNARE complex, vesicle membrane and plasma membrane, Cpx1 and Syt1 lock the fusion complex into a prefusion state [206]. 3) In the step of Ca^2+^-triggered vesicle fusion, the fusion loop of Syt binds to Ca^2+^ and inserts into the anionic membrane [252, 254, 359, 360]. Meanwhile Syt1 interacts with the anionic lipid via the polybasic region, and with the SNARE complex via the primary interface, thereby inducing fusion pore opening via the *cis*-SNARE complex formation [240, 268, 336, 359, 361]. 4) After the cargo is released through the fusion pore, the *cis*-SNARE complex is disassembled by NSF and α-SNAP in response to ATP hydrolysis [362–367]. The disassociated SNARE proteins are then available for a new round of vesicle formation and fusion. In summary, SNARE-mediated vesicle fusion is highly dynamical and precisely controlled by Syt, Cpx, Munc13, Munc18 and others, including probably unidentified regulators, and dysfunction of any fusogenic protein in each substep of vesicle fusion could result in the development of a wide-range of membrane fusion related diseases.

## Dysfunctions of vesicle trafficking-related proteins and diseases

Vesicle trafficking and fusion between organelles, as an important cell signaling pathway, maintain intracellular material exchange and intercellular signal transmission. Vesicle trafficking disorder can lead to a series of diseases, such as nervous system diseases, respiratory system diseases, immune system diseases, diabetes and so on [368, 369]. Pathological changes include the obstruction of vesicle trafficking and over secretion of vesicle cargo, so that reverse manipulation of vesicle trafficking could be the potential therapy for vesicle trafficking disorder related diseases. Since numerous proteins are involved in vesicle trafficking, and it is not realistic to discuss all the dysfunctions of these proteins related diseases. In this section, we only focus on the dysfunction of fusogenic proteins related diseases, because the molecular mechanism of membrane fusion between vesicle and cell membrane has been well elucidated, and manipulating these proteins are less likely to have an impact on the early steps of vesicle trafficking. Moreover, recent study shows that Ca^2+^-triggered membrane fusion can be manipulated to prevent the airway mucus occlusion in mice with asthma [370]. It suggests that the membrane fusion process can be controlled pharmacologically and targeting to the vesicle trafficking-related protein could be a novel therapeutics strategy for the drug development across a wide range of therapeutic areas.

### Respiratory airway diseases

In the surface epithelium of intrapulmonary airway system, the secretory cells express and secrete mucins to extracellular airway lumen [371]. In the healthy state, mucin secretion is at low baseline level which is critical for the defense and clearance of inhaled particle and pathogens. When allergic mucus metaplasia occurs, the mucus is greatly increased in production and accumulated in the airway lumen. Mucus hypersecretion is a major cause of airway obstruction in the pathophysiology of airway related diseases, such as asthma and cystic fibrosis.

Up to now, most drugs are developed to reduce inflammation or expand the airways [371, 372], but the most serious issue is airway obstruction caused by mucus accumulation. The drugs approved to deal with mucus are targeted on mucin production, mucus hydration or mucus digestion [371, 373, 374]. Although some of available drugs can improve lung health in clinical trials [375], the limitations on their applications are also obvious. For example, treatments that inhibit mucin over production failed to reduce the mucin storage in airway epithelial cells in a clinic trial [376]. Moreover, treatments that reduce mucus accumulation via digesting DNA polymers in the mucus layer are only suitable for cystic fibrosis but not for other obstructive lung disease [377]. Finally, cystic fibrosis transmembrane conductance regulator (CFTR) modulator therapy is restricted to the patients with certain genetic mutations [378].

**Fig. 7:**
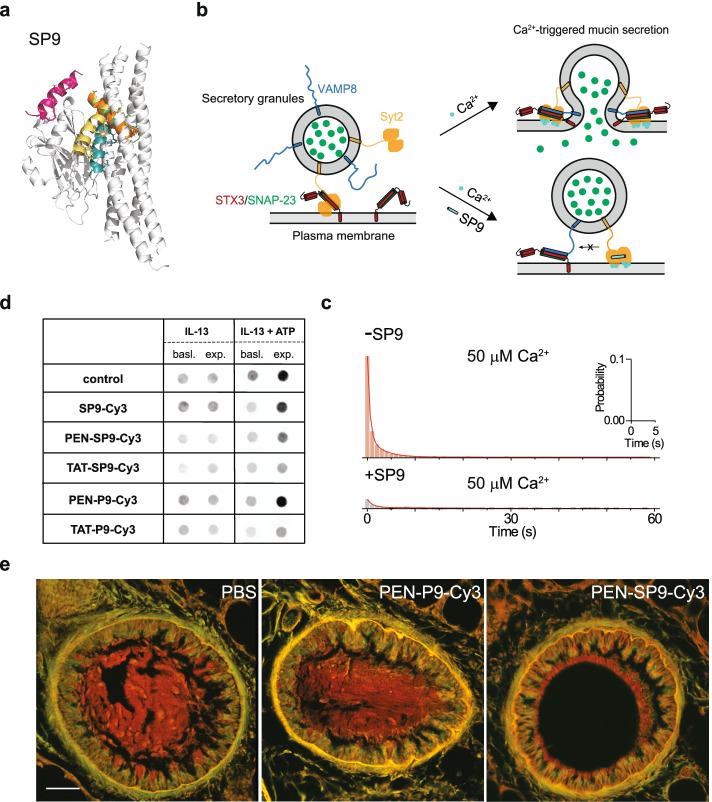
Inhibition of mucus hypersecretion by a stapled peptide. **a** Molecular dynamics simulations of SP9-Syt1-C2B complex superimposed on the structure of the primary interface (gray). **b** The therapeutic strategy to inhibit mucus hypersecretion. Binding of SP9 to Syt2 blocks the interaction between Syt2 and SNARE complex, thereby disrupts the Ca^2+^-triggered mucus hypersecretion. **c** SP9 inhibits Ca^2+^-triggered fusion probability in a reconstituted system. **d** SP9 inhibits mucin secretion from airway epithelial cells. Image represents western blots for MUC5AC on apical surface of untreated HAECs, or HAECs treated with different peptides. **e** SP9 inhibits mucin secretion and airway occlusion in mice. Images represents transverse sections of bronchial airways of mice with asthma pre-treated with PBS or peptides. Panels a, c-e, are adapted from [370]

A novel strategy for reducing mucus accumulation is to inhibit mucus hypersecretion (Fig. 7a-b). Mucus hypersecretion is mediated by the SNARE proteins and the Ca^2+^ sensor Syt2. In stimulated mucin secretion, when agonists like ATP bind to purinergic receptors coupled to G proteins in the plasma membrane, inositol triphosphate (IP3) is generated by phospholipase C. Then, Ca^2+^ is released from ER via the activated IP3 receptor. In turn, Ca^2+^ binds to Syt2 and accelerates mucin secretion [371].

Previous studies demonstrated that the primary SNARE-Syt interface is a genuine and specific interface and conserved in all species [240]. Disruption of the primary interface by mutations abolished fast synchronous release in cultured neurons and greatly reduces the Ca^2+^-triggered fusion probability in single-vesicle fusion experiments. Based on the structure of the SNARE-Syt complex, an engineered stapled peptide SP9 derived from the SNAP-25A fragment was designed to compete with the SNARE-Syt interaction (Fig. 7b). In a reconstituted fusion system, SP9 substantially reduced the Ca^2+^-triggered membrane fusion but has only mild effect on Ca^2+^-independent membrane fusion (Fig. 7c). Moreover, after being delivered into airway epithelial cells via conjugating to a cell penetrating peptide, SP9 strongly suppressed stimulated mucin secretion, but not incurred toxicity because of the mild effect on basal mucin secretion (Fig. 7d). Finally, short term treatment of mice with aerosolized SP9 resulted in substantial peptide uptake into airway epithelial cells, and markedly reduced methacholine-stimulated, Ca^2+^-triggered mucin secretion and airway mucus occlusion, but not baseline mucin secretion (Fig. 7e). Thus, disruption of the SNARE-Syt2 mediated mucus hypersecretion could be a new therapeutic strategy for obstructive lung disease.

Many approved therapeutic peptides have shown a low rate of immunogenicity in clinical trials [379–381], so SP9, as a therapeutic compound, is unlikely to cause any immune response. However, considerable optimization and pharmacokinetic studies are required before moving to clinical trials, including maximizing cellular uptake, improving intracellular stability, and increasing potency. In summary, stimulated membrane fusion processes, such as neurotransmitter release or mucin secretion, can be manipulated pharmacologically by compounds that disrupt the interaction between the fusogenic proteins and Ca^2+^ sensors. From a broader perspective, it suggests that targeting to fusogenic proteins such as Syt, have therapeutic value in the pathophysiology of common membrane fusion disorder related diseases.

### Neurological diseases

#### Neurodegenerative diseases

In nervous system, almost all the neurodegenerative diseases are associated with dysregulation of synaptic vesicle trafficking. Abnormal expression or dysfunction of fusogenic proteins results in series of diseases in neuronal system. Accumulation of Aβ in neurons is one of the hallmarks for AD patients. Besides, in AD patients and mice, oligomerized Aβ inhibited the SNARE complex formation via competitively binding to STX1A with VAMP2, leading to the defects in neurotransmission and cognitive function [382, 383]. In addition, in AD brain, it was found that expression level of Syt1 was significantly reduced in the regions related to cognitive and memory functions by the experiments of mass spectroscopy and western blot [384, 385]. And one microRNA miR-34c in AD mice negatively regulated Syt1 expression, while miR-34c antagomir markedly increased the brain levels of Syt1, rescuing synaptic and memory deficits [386]. Moreover, in AD mice, deletion of Munc18-1 displayed dysregulation of tau phosphorylation, neurofibrillary tangle accumulation and alterations of the ubiquitination state which are also the hallmarks of AD [387].

Parkinson’s disease (PD) is characterized by the accumulation of misfolded and fibrillary forms of α-synuclein (α-syn) in neurons. α-syn plays a role in the formation of SNARE complex as a chaperon via binding to N-terminal of VAMP2, and regulates vesicle trafficking and synaptic transmission [388]. Whereas pathotype mutations of α-syn from rare inherited PD inhibit vesicle fusion and neurotransmitter release via binding to anionic membrane [389–391].

Moreover, it was reported that modification of VAMP2 with a “non-cleavable” N-terminal ubiquitin substrate could lead to progressive impairment of synaptic transmission at the neuromuscular junction followed by the degeneration of motor nerve terminals (i.e. ALS) [392].

#### Neuropsychiatric diseases

For schizophrenia disease, expression levels of SNAP-25A and Cpx1 are reduced [393–395], while the interaction between Cpx1 and SNAREs increases in schizophrenia patients [396]. Moreover, it is observed that Ser14 phosphorylated STX1 is decreased in post mortem prefrontal cortex of schizophrenia patients [397], although the function of phosphorylation of STX1 was still unclear. Some studies suggested that phosphorylation of STX1 was required for the SNARE complex formation because it could enhance the binding to SNAP-25 and Munc18-1 [398]. But in other studies, phosphorylation of STX1 inhibited the probability of vesicle exocytosis in neuronal cells, because of its function in regulating the N-terminal interaction with Munc18-1, which promotes vesicle docking at the plasma membrane [399]. Last, one study revealed that phosphorylation of STX1 reduced vesicle exocytosis from slowly releasable pool without effect on the readily releasable pool [400].

In the plasma samples of bipolar disorder (BD) patients, the Syt7 mRNA level was significantly reduced, and in Syt7 knockout mice, mood cycling symptoms of BD was observed [401, 402]. As a result of Syt7 defects, the activity of GluN2B-NMDARs was attenuated by disruption of spontaneous glutamate release, which induced mania-like behavioral abnormalities [403]. After being treated with clinical BD drugs such as olanzapine, which could induce a significant extracellular release of glutamate in mice by inhibiting the activity of the catabolic enzyme D-aspartate oxidase, it could efficiently prevent the behavioral abnormalities of Syt7 knockout mice [404].

#### Other neurological diseases

In other cases, hypersecretion of neurotransmitter and sensory neurochemicals, such as substance P, calcitonin gene‐related peptide, glutamate and so on, could cause neuropathic by conveying pain sensation from the peripheral to the central nervous system including the spinal cord [405]. Botulinum neurotoxin type A (BoNTA) is known to cleave SNAP‐25, and prevent synaptic vesicle fusion. In a double‐blind placebo‐controlled trials, the efficacy of BoNTA was confirmed for the prevention of headaches in chronic migraine patients [406]. Although BoNTA is a potential drug candidate, its therapeutic use is limited because of its high toxicity. It was designed a small peptide drug candidate DD04107 (Palmitoyl-EEMQRR-NH2) that to substituted BoNTA by destabilizing the SNARE complex [407, 408]. This peptide was also found to interact with Syt1-C2B domain at the primary interface selectively, suggesting that Syt1 is a potential new analgesic target [407]. Similarly, suppressing Syt1 expression by stimulating acupoints of rats with electroacupuncture could attenuate neuropathic pain, indicating Syt1 involves in the development of neuropathic pain [409].

Numerous mutations of SNAREs and Syt1 were found in patients with neurodevelopmental disorders, including developmental and epileptic encephalopathies (DEEs), and it was likely due to the mutants caused abnormality in neurotransmitter release [410–413]. For example, most of the mutations on SNARE proteins occurred at the SNARE motif, which disrupt the SNARE complex formation [410, 412, 413]. In addition, mutations of SNAP-25 (K40E, V48F and D166Y) on the residues in the primary interface, impaired SNARE-Syt1 interaction [413]. Moreover, mutations of STX1 on N-terminal peptide and Habc domains, interfered the interaction with Munc18-1, while mutations of STX1 on the C-terminal transmembrane domain, impaired its location at plasma membrane [410]. Finally, mutations (D304G, D366E, I368T, N371K) of Syt1 occured in Ca^2+^ binding site and fusion loop, could severely interfere Ca^2+^-evoked neurotransmitter release [411].

### Endocrine system disease

The main cause of type 2 diabetes mellitus (T2DM) is dysfunction of insulin secretion in pancreatic β-cells and insulin resistance in skeletal muscle, liver and fatty tissue [414]. The glucose homeostasis is regulated by insulin, which raises glucose uptake into skeletal and fat cells from blood. In response to insulin, glucose transporter 4 (GLUT4) is translocated to the plasma membrane by fusion with GLUT4 storage vesicles (GSVs), and the failure of this process is an early step in the development of insulin resistance and T2DM [415]. GSV fusion with the plasma membrane is regulated by SNAREs (STX4, SNAP-23 and VAMP2) [416]. The insulin receptor catalyzes phosphorylation of Munc18-3 at Tyr219 and Tyr521 and resulted in Munc18-3 switching the binding from STX4 to a double C2-like domain-containing protein-β (DOC2β) [417, 418]. Inhibition of GSV fusion induced the insulin resistance in muscle cell, which in turn might interfere insulin secretion in pancreatic β-cells [419]. Furthermore, the insulin secretion is also regulated by SNARE and other fusogenic proteins. Based on the resources of secretory granules that are pre-docked or not, insulin secretion exhibits a biphasic pattern [420]. For the secretion of pre-docked granules, it is a transient but rapid process that regulated by SNAREs (STX1A, SNAP-25 and VAMP2), Munc18-1, Munc13-1 and Syt7, and triggered by Ca^2+^, while for the secretion of non-docked granules, it is a 5 ~ 10 min persisting release process that regulated by SNAREs (STX3, SNAP-25 and VAMP8), and Munc18-2 [421, 422]. In T2DM mice, treating with fibroblast growth factor 21 could promote the expression of SNAREs to elevate insulin secretion, thereby maintaining insulin homeostasis and pancreatic β‐cell function [423]. Therefore, manipulation of GLUT4 translocation and insulin secretion could be considered as a potential treatment of T2DM.

### Immune system diseases

Familial hemophagocytic lymphohistiocytosis (FHL) is a genetically heterogeneous disease with defective cytotoxicity caused by dysfunction of cytolytic granules secretion at the immunological synapse. The lytic granules secretion is mediated by STX11, SNAP-23, VAMP7, Munc18-2, and Munc13-4, and mutations of these proteins cause defective immune function [424–428]. In FHL3 patients, various mutations of Munc13-4 were found, such as various truncations of Munc13-4 caused by different splice sites, nonsense mutations, and frameshift. All the mutations of Munc13-4 were found to interfere the priming step of cytolytic granules secretion [428]. In addition, mutations of STX11 and Munc18-2 were associated with FHL4 and FHL5, respectively [424, 429]. It was found that in FHL4 the defect in the interaction between STX11 and Munc18-2 was caused by mutations of STX11 (R4A, L58P), while mutations of Munc18-2 (E132A, P477L) in FHL5, disrupted cytolytic vesicle fusion [430, 431]. Moreover, in FHL4 patients, the nonsense mutations of STX11 (W382X, Q268X) led to the lack of the C-terminal cysteine-rich motif, resulting in its failure of localization on plasma membrane [432]. While in FHL5 patients, lack of Munc18‐2 also resulted in the failure of localization of STX11 to the plasma membrane of cytotoxic T-lymphocytes, suggesting the localization of STX11 onto plasma membrane depends on Munc18‐2 [433].

Histamine, as a key factor in allergic disease, is stored in histamine granules in mast cells and degranulated after allergen-mediated cross-linking of immunoglobulin E on mast cells. Histamine secretion from mast cell is mediated by SNAREs (STX3, SNAP-23, VAMP8), Munc18-2, and Munc13-4 [434–436]. Atopic dermatitis is a chronic inflammatory dermal disease caused by histamine with symptoms including inflammation, itching, and dry skin. 1-Iodohexadecane treatment significantly decreased VAMP8 expression level, therefore reduced histamine release in mast cells and alleviates the symptoms for the mice with atopic dermatitis [437]. In another study, peptides derived from various SNARE motifs, such as a N-terminal-mimicking peptides derived from VAMP8, was investigated for their potential inhibitory effects against the formation of SNARE complex and mast cell degranulation [438]. Thus, inhibiting the mast cell degranulation could be a potential strategy to prevent histamine secretion and to alleviate atopic dermatitis symptom.

## Conclusions and perspectives

Intracellular vesicle trafficking builds the communication network inside cells, which is important for maintaining the homeostasis of intracellular organelles. Although the function and mechanism of vesicle trafficking-related protein has been intensively investigated, the questions that how these supramolecule of the protein complex works together remain to be answered. For example, how specific phosphoinositide levels are controlled on CCPs during vesicle formation? Second, how do Syt1, Cpx1 and SNAREs cooperate to inhibit vesicle fusion in the absence of Ca^2+^? Third, how do Munc13, Munc18 and SNAREs cooperate to ensure the proper SNARE complex formation? Last but not least, what is the arrangement of supramolecule of the fusogenic proteins complex around the fusion pore in response to Ca^2+^? Understanding how these key proteins correctly coordinate during vesicle trafficking can help us to interpret how pathogenic factors disrupt the precisely regulated pathway. For example, in airway system, different Munc18 isoforms mediate baseline and stimulated airway mucin secretion, but the molecular mechanism underneath is still unclear. Further research into the pathology and mechanism of how Munc18 and Syt2 cooperate to induce stimulated mucin secretion, would aid in the development of new therapeutic approaches to selectively manipulate stimulated mucin secretion for obstructive pulmonary disease.

Dysfunctions of membrane fusion are closely related to the occurrence of various diseases, although the underlying molecular mechanism are not well understood. Current data suggests that the fusogenic proteins could serve as a starting-point of therapeutic target for vesicle fusion disorder related diseases, and manipulating vesicle fusion could be an entirely new therapeutic strategy for drugs development. Up to now, several potential drugs show great efficacy to treat corresponding diseases in experiment model, such as SP9, and small VAMP8 peptide, and DD04107 that could inhibit neurotransmitter release by destabilizing the SNARE complex, is currently active in phase II clinical trials for neuropathic pain. Considering the mechanism of SNARE mediated vesicle fusion is conserved and universal in different organelles and tissues, inhibition or manipulation of membrane fusion processes would have wide-ranging applications for the therapy of membrane fusion disorder related diseases, including obstructive lung disease, viral-host membrane entry and virus exocytosis. However, it is worth noting that due to the highly conservative mechanism, special attention should be paid to whether the related drugs may have extensive toxicity in multiple organs and tissues. In the follow-up drug research, more efforts might be needed to improve the organ selectivity and targeting of drugs.

## Data Availability

Not applicable.

## Abbreviations

AAA: ATPases associated with diverse cellular activities
AD: Alzheimer's disease
ALS: Amyotrophic lateral sclerosis
AP1: Adaptor protein 1
AP2: Adaptor protein 2
Arf1: ADP-ribosylation factor 1
BARS: BrefeldinA-ADP-ribosylated substrate
BD: Bipolar disorder
BET1: Blocked early in transport 1
BNIP1: B-cell lymphoma-2 interacting protein 1
BoNTA: Botulinum neurotoxin type A
CAPS: Caspase
CC: Coiled-coil
CCP: Clathrin-coated pit
CCV: Clathrin-coated vesicle
CFTR: Cystic fibrosis transmembrane conductance regulator
CGN: *Cis*-Golgi network
COG: Conserved oligomeric Golgi
Co-IP: Co-immunoprecipitation
COPII: Coat protein complex II
COPI: Coat protein complex I
CORVET: Class C core vacuole/endosome tethering
cryo-EM: Cryo-electron microscopy
Dab2: Disabled 2
DEEs: Developmental and epileptic encephalopathies
DOC2β: Double C2-like domain-containing protein-β
EEA1: Early endosomal antigen 1
ENTH: Epsin N-terminal homology
EPG5: Ectopic P granules protein 5 homolog
eps15R: Eps15 related
ER: Endoplasmic reticulum
ERES: ER exit site
ERGIC: ER-Golgi intermediate compartment
FBP17: Formin-binding protein 17
FCHO1/2: Fer/CIP4 homology domain only protein 1/2
FHL: Familial hemophagocytic lymphohistiocytosis
FYCO1: FYVE and coiled-coil domain-containing protein 1
FYVE: Fab 1, YOTB, Vac 1, and EEA1
GAP: GTPase-activating protein
GARP: Golgi-associated retrograde protein
GBF1: Guanine nucleotide exchange factor for Arf1
GEF: Guanine exchange factor
GGA: Golgi-localized, gamma-adaptin ear homology, Arf-binding protein
GS27: Golgi SNAREs of 27 kDa
GS28: Golgi SNAREs of 28 kDa
GSVs: GLUT4 storage vesicles
HDS1: Homology downstream of Sec7d-1
HOPS: Homotypic fusion and protein sorting
IP3: Inositol triphosphate
KIF1C: Kinesin family member 1C
LC3B: Microtubule-associated proteins 1A/1B light chain 3B
PD: Parkinson’s disease
PH: Pleckstrin homology
PI(4,5)P2: Phosphatidylinositol-4,5-bisphosphate
PI3P: Phosphatidylinositol-3-phosphate
PI4P: Phosphatidylinositol-4-monophosphate
PLD2: Produced by phospholipase D2
PLEKHM1: Pleckstrin homology domain-containing family member 1
PRD: Proline-rich domain
PTM: Post-translational modifications
Q-SNARE: Glutamine-containing SNAREs
RIM: Rab-interacting molecule
R-SNARE: Arginine-containing SNAREs
SM: Sec1/Munc18
SNAP: Soluble NSF attachment protein
SNAP-25A: Synaptosomal-associated protein 25 kDa A
STX5: Syntaxin-5
T2DM: Type 2 diabetes mellitus
TGN: *Trans*-Golgi network
TRAPPI: Transport protein particle I
t-SNARE: Target membrane-SNARE
USE1: Unconventional SNARE in the ER1
VAMP2: Vesicle associated membrane protein 2
Vps45: Vacuolar protein sorting homolog
v-SNARE: Vesicle-SNARE
VTI1A: Vesicle transport through interaction with t-SNAREs homolog 1A
VTI1B: Vesicle transport through interaction with t-SNAREs 1B
ZF: Zinc finger
α-syn: α-Synuclein
μHD: μ Homology domain
